# Introducing the Tele-OCS: Preliminary evidence of validity for a remotely administered version of The Oxford Cognitive Screen

**DOI:** 10.12688/healthopenres.13291.3

**Published:** 2025-07-09

**Authors:** Sam S. Webb, Chloe Carrick, Andrea Kusec, Nele Demeyere

**Affiliations:** 1 Nuffield Department of Clinical Neurosciences, University of Oxford, Oxford, Oxfordshire, OX3 9DU, UK; 2 Experimental Psychology, University of Oxford, Oxford, Oxfordshire, OX2 6GG, UK

**Keywords:** remote assessment, teleneuropsychology, cognitive screening, cognitive assessment

## Abstract

**Background:**

Remote cognitive assessments are increasingly used with the rising popularity of teleneuropsychology. Here, we evaluated the performance of the remotely administered Oxford Cognitive Screen (Tele-OCS) compared to in-person administration in adult stroke survivors.

**Methods:**

40 stroke survivors ( *M* age = 69.30, *SD* = 10.44; sex = 30% female) completed in-person and remote versions of the OCS on average 30 days apart, with different trained examiners. The order of administration was counterbalanced. Cohen’s *d* estimates were used to compare performance between modalities.

**Results:**

We found that the proportion of OCS subtasks impaired did not differ across modalities ( *d* <.001). With regards to raw subtask scores, only the picture naming subtask and executive score from the trail making subtask were found to be statistically different across modalities, though raw differences were minimal (<1 point difference on average). These statistical differences did not affect impairment classifications.

**Conclusions:**

The Tele-OCS classified cognitive impairments in a comparable way to the in-person version. The validation of the Tele-OCS allows for remote assessment to increase accessibility and pragmatically aid in addressing the clinical need for stroke-specific cognitive screening in a wider population.

## Plain Language Summary

Background Commonly after a stroke, people have changes in specific thinking abilities (cognition). These may include difficulties with understanding, reading, writing, remembering, spatial awareness and planning activities. The Oxford Cognitive Screen (OCS) was made to briefly assess these stroke-specific changes in thinking abilities. The OCS is now well known in the stroke clinical community, with >1200 licensed stroke settings. During the pandemic the OCS was administered remotely (phone or video call etc.), but there was no evidence to support the use of this modality. Here, we evaluated the performance of the Oxford Cognitive Screen administered via phone and video call (Tele-OCS) compared to in-person administration in adult stroke survivors. Methods 40 stroke survivors (M age = 69.30, SD = 10.44; sex = 30% female) completed in-person and remote versions of the OCS approximately 30 days apart, with different trained examiners. The order of administration was counterbalanced. Statistical estimates were used to compare performance between modalities. Results We found that the proportion of OCS subtasks impaired did not differ across modalities, meaning individuals were not more impaired on one modality over the other. With regards to raw subtask scores, only the picture naming subtask and executive score from the trail making subtask were found to be statistically different across modalities, though raw differences were minimal (<1 point difference on average). These statistical differences did not affect impairment classifications. Conclusions The Tele-OCS classified mental ability impairments in a comparable way to the in-person version. The validation of the Tele-OCS allows for remote assessment to increase accessibility and pragmatically aid in addressing the clinical need for stroke-specific cognitive screening in a wider population.

## Introduction

Whilst improved stroke care in hospital has led to a decrease in stroke-related mortality, the number of individuals living with the long-term complications of stroke has risen ( Amy & Kapral, 2019). There are greater than 100,000 individuals affected by stroke each year ( King *et al.*, 2020). The number of strokes in Europe is projected to rise by 34% by 2035 ( King *et al.*, 2020). Post-stroke cognitive impairment is common, with cognitive deficits experienced by almost all stroke survivors early after stroke ( Demeyere *et al.*, 2016; Esmael *et al.*, 2021; Merriman *et al.*, 2019; Sexton *et al.*, 2019), and can negatively impact rehabilitation outcomes and post-stroke quality of life ( Mole & Demeyere, 2020; Nys *et al.*, 2006). Clinical guidelines have called for the early screening of post-stroke cognition using valid and reliable tools ( Intercollegiate Stroke Working Party, 2016; Intercollegiate Stroke Working Party, 2023; Quinn *et al.*, 2021; Rudd *et al.*, 2017).

Teleneuropsychology, which includes the remote delivery of neuropsychological assessments alongside therapy interventions, has long existed alongside the technology to support it ( Munro Cullum *et al.*, 2014). The use of remotely administered cognitive tests dramatically increased following the onset of the COVID-19 pandemic, with many healthcare professionals shifting the modality of assessment to telephone or video conferencing ( Chapman *et al.*, 2020; Fox-Fuller *et al.*, 2022; Hammers *et al.*, 2020; Webb *et al.*, 2022). This expansion in remote neuropsychological assessment led to the adaptation of widely used cognitive assessment tools for remote use. However, research on the validity of these adaptations has lagged behind. Widely-accessible remote cognitive assessment tools were underdeveloped at the onset of COVID-19, with low numbers of freely available standardized and validated versions of cognitive screening tools ( Webb *et al.*, 2022). Tools such as the telephone Montreal Cognitive Assessment, (MoCA; Benge & Kiselica, 2021; Katz *et al.*, 2021; Pendlebury *et al.*, 2013), the Telephone Interview for Cognitive Status ( Brandt *et al.*, 1988), and the 26-point telephone version of the Mini Mental State Examination (MMSE; Newkirk *et al.*, 2004) were developed for screening for general dementia, not for early stroke-specific cognitive domains typically affected by stroke (e.g., visuospatial neglect, apraxia, aphasia etc.; Demeyere *et al.*, 2015; Demeyere *et al.*, 2016). Most of these tools also require a paid license, or paid training certification to gain access and use the materials.

Whilst there has been an increase in remote cognitive assessments, no stroke specific screen was validated for remote use. Domain-specific screening allows health professionals to identify specific cognitive profiles, with both cognitive impairments, as well as domain-specific strengths. This allows for targeted and personalized rehabilitation and discharge planning, instead of providing a broad brush overall cognitive picture in binary terms (impaired/un-impaired classification). The Oxford Cognitive Screen (OCS) is a stroke-specific, in-person screen of cognitive impairment, consisting of 11 normed tasks which assess functioning in the domains of memory, language, praxis, executive functioning, and attention ( Demeyere *et al.*, 2015). The OCS provides both raw continuous performance scores per subtask as well as subtask-specific impairment classification compared normative data, and a total proportion of subtasks impaired score. The visual snapshot reports the performance across five cognitive domains. The OCS has been shown to be more sensitive to detecting post-stroke cognitive impairment than both the MoCA and MMSE ( Demeyere *et al.*, 2016; Mancuso *et al.*, 2018). Since its original English publication with UK norms, the OCS has seen several cultural and language adaptations and translations, normed and validated for use in different countries ( Hong *et al.*, 2018; Huygelier *et al.*, 2020; Kong *et al.*, 2016; Mancuso *et al.*, 2016; Ramos *et al.*, 2018; Robotham *et al.*, 2020; Valera-Gran *et al.*, 2019; Valério *et al.*, 2022) and is now widely used across the globe.

The current study aimed to validate a remote version of the Oxford Cognitive Screen ( Demeyere *et al.*, 2015) that can be administered via telephone or videoconferencing. Interim provisional guidance for administration of a remote version of the OCS was released in May 2020 ( Raymer, 2020), following frequent requests to the authors. Indeed, there is evidence to suggest that remote versions of the OCS were administered throughout the COVID-19 pandemic ( Webb *et al.*, 2022). However, no formal validation of a remote delivery format of OCS had been conducted until now.

### Study purpose

The purpose of this study is to compare classification of impairment on the Oxford Cognitive Screen when administered in-person versus remotely (via telephone or videoconferencing) with adults at least 6-months post-stroke. We do not expect any sex differences to affect results. Our pre-registered hypotheses ( Webb *et al.*, 2022) were as follows and any deviations from the pre-registration are reported transparently:

1. Each OCS subtask continuous performance score will have moderate to strong associations across in-person and remote versions (ICC ≥ 0.50);
2. The sensitivity and specificity values of the in-person and remote version of the OCS will be approximately equitable.

## Methods

We report how we determined our sample size, all data exclusions, all manipulations, and all measures in the study ( Simmons *et al.*, 2012). Approval for the study was granted by the Medical Sciences Interdivisional Research Ethics Committee (First approved July 2021, amendment approval Jan 2022, REC REF: R58224/RE001). We adhere to the STROBE cross-sectional study checklist ( von Elm *et al.*, 2007).

### Participant identification

Eligibility criteria were: 1) history of confirmed stroke; 2) 18 years of age or older at time of stroke; 3) capacity to consent to research; 4) able to remain alert for at least 20 minutes; and 5) spoke and understood sufficient English. Participants were excluded if they had hearing, language, or visual impairments (not including visual neglect) that would not allow for remote or in-person assessment outside of reasonable adjustments. Any hearing difficulties that became evident whilst testing were overcome by repeating a single instruction twice.

### Study design and sample size

We used a prospective cross-sectional within-subjects design. Due to the standard practice of inpatient stroke care within Oxfordshire, most stroke survivors admitted to a hospital for stroke will complete, or partially complete, the OCS. We recruited chronic stroke survivors in the UK between November 2021 to August 2022. All participants completed the OCS when originally admitted to hospital, separate to this study. We contacted long-term stroke survivors from the OXCHRONIC ( Kusec *et al.*, 2023) study who had recently (within 30 days at time of contact) completed the tele-OCS as part of the OXCHRONIC protocol. The OXCHRONIC population were at minimum 2 years post-stroke with an average of 4.5 years. Additionally, we asked all participants from the Oxford Cognitive Screening programme, who had just (at time of being contacted) completed an in person 6-month follow up paper OCS ( Demeyere *et al.*, 2015; Milosevich *et al.*, 2023), to take part. We aimed to get both the OCS and Tele-OCS completed within 30 days of each other, but this was not always possible. Inclusion criteria for both the Oxford Cognitive Screening programme and OXCHRONIC are very inclusive and aligned with the current inclusion criteria. This means the sampling approaches originally used have minimal bias (except bias of those who are willing to participate), and the underlying samples are representative of the UK stroke population. We acknowledge that by study design, participants will be exposed to the same test twice and no matter the order of administration (remote or in-person) they may improve on the second test. The study design is typical and is shared with multiple other comparison studies of modality equivalence ( Brown *et al*., 2024; Duran *et al*., 2024; Morrissey *et al*., 2024). We used two methods to address possible practice effects: We ensured that different sets of the sample receive opposite administration order (Tele-OCS vs. OCS) and different versions (A or b). We further used group statistics to examine if participants improve on their second administered test.

Sample size was pre-determined in our pre-registration at a minimum of 30 participants to detect a two-way random effects single unit agreement based intraclass correlation coefficient (ICC) of greater than .50, with 90% power, and an alpha of 0.05. Recruitment was conducted irrespective of participant demographics, including sex, and how these may relate to OCS subtasks, as this was not a main aim of the study. Sex was determined via medical records of sex-assigned at birth when recruiting participants.

### Changes from pre-registration

This project was originally pre-registered at the start of data collection (3 participants had taken part). The preregistered analysis plan was inspired by ICCs between OCS parallel versions A and B in the original OCS normative article ( Demeyere *et al.*, 2015), though once data was collected, it became clear that this was not the best approach for the current data that was observed. The small variation of scores observed in the subtasks meant the raw continuous scores ICCs between in-person and remote subtest comparisons were not appropriate. We instead opted to shift towards test of difference between modalities, to retain consistent statistical power. The sample size calculations remained the same as we were similarly powered for equivalent comparisons via difference based *t*-tests. We present ICC analyses in extended data.

In line with the clinical relevance of cognitive screening, we instead used the binary impairment classifications rather than continuous performance scores to determine if deviations between in-person and remote assessments would change interpretation of overall impairment status and hence interpretation of the assessment. We re-ran the analysis after collecting further participants (n = 9) who completed only OCS version A both remotely and in-person due to the timeline in which we changed our analysis plan. We included these participants in the final dataset. Power to detect effects was unchanged when adapting only impairment classification analysis over continuous scores.

### The Oxford Cognitive Screen (OCS)

The OCS is usually administered in-person by health care professionals using pen and paper and takes around 15 to 20 minutes to administer ( Demeyere *et al.*, 2015). The task can be administered at bedside but is best administered at a table. The OCS requires a testing booklet, examiner forms, and participant pack (see www.ocs-test.org).

The OCS tasks include:

- a picture naming task
- a semantics task (pointing to images based on verbal instruction)
- an orientation task (verbal and multiple choice question response permitted)
- a basic visual field assessment (the examiner holds hands up in upper and lower left and right visual quadrants)
- a sentence to read
- numbers to write
- calculations to complete verbally (or via multiple choice if the participant has expressive problems)
- a spatial inattention task
- a gesture imitation task (the examiner presents gestures with hands and fingers and the participants is instructed to mirror these)
- delayed verbal and episodic recognition of the sentence and previously presented images
- a symbol trail making test (two shape based lines and a mixed trail).

All subtasks have continuous performance scores and are classified as impaired/not impaired based on normed cutoffs ( Demeyere *et al.*, 2015). A proportion of tasks impaired score can also be calculated (the summed number of subtasks impaired divided by the number of subtasks attempted). Subtasks belong to five separate cognitive domains; language (picture naming, semantics, sentence reading), number (number writing and number calculation), praxis (gesture imitation), memory (orientation, episodic memory, delayed recall and recognition, and sentence recall), and executive attention (trail making test and spatial inattention test).

### The Tele-OCS: a remotely administered version of the Oxford Cognitive Screen

The remote version of the OCS is an adaptation of version A of the OCS. Version A is currently the only version used here, as this was the form used in OxCHRONIC which did not repeat testing with version B. A version B will be available in the future, but is not used in the current study. Administration of the Tele-OCS can be conducted via telephone or video-call. Before the appointment, participants are sent a single testing booklet through the post containing the necessary stimuli to complete the OCS remotely. Participants are instructed not to open the pack before the phone or videocall appointment and are asked to ensure they sit at a table for the duration of the session and have a pen or pencil to hand. The assessor keeps a copy of the remotely sent materials in front of them to jointly follow along with the participant through each subtask. During the session, participants are given specific instructions (provided in the user manual for the examiner) on how to complete each subtask of the Tele-OCS, including the relevant page to turn to in their testing booklet, when to pick up and put down their pen or pencil, and which way to position their booklet on the table. If completing the Tele-OCS via telephone call, participants were instructed to put the telephone on speaker mode, allowing participants freedom to use both hands to complete the subtasks. Upon completion of the assessment session, participants were asked to place the testing booklet into a pre-paid addressed envelope and return it to the assessors via the post. Where necessary, facilitation of page turning and reinforcing assessor instructions was supported by a carer or family member.

Several differences exist between the in-person and remote version of the OCS. Given that the praxis and visual field subtasks require the participant to be in full view of the assessor, these are not assessed as part of the Tele-OCS. To assess constructional praxis, the Tele-OCS instead contains a figure-copy task, requiring participants to copy, and immediately recall, a complex figure. The figure was taken from the OCS-Plus assessment, under the same copyright as the OCS ( Demeyere *et al.*, 2015). The spatial attention task was adapted for remote administration by giving two practice rounds, instead of one, to complete before the main task. This was put in place to allow the examiner to gauge whether the participant has understood the instructions, without being able to see the practice trials. On the first round, participants practice a short version of the spatial attention subtask, where they are presented with six line-drawings of hearts, three of which contain a gap on either the left or right-hand side. Participants are instructed to only cross out the complete hearts on the page. On the second round, participants verbally state which of these hearts have a gap and which ones are complete. This additional practice round allows the assessor to evaluate whether the participant has understood the instructions before they proceed to the main task.

 Finally, adaptations were made to the remote version of the OCS trail making task. When this subtask is administered in-person, participants are shown a completed example by the assessor which they can refer to throughout the duration of the task. As this is not possible through remote administration, instructions have been printed at the top of the practice task and the main task in the remote testing booklet. The materials for the Tele-OCS and the manual for administration are freely available for publicly funded clinical and research use in the same way as the in-person OCS. The Tele-OCS is licensed through Oxford University Innovations Health Outcomes ( https://innovation.ox.ac.uk/outcome-measures/the-oxford-cognitive-screen-ocs/).

We timed the duration of the Tele-OCS administration for a portion of participants only ( *n* = 18). We additionally recorded any pragmatic information regarding the optimal assessment of the Tele-OCS. This involved recording unsolicited spontaneous feedback from participants on the Tele-OCS after administration.

### Procedure

Once participants from the Oxford Cognitive Screening programme were identified as having just (within 30 days) completed an in-person OCS in their homes, we asked if they would complete the Tele-OCS. For those who agreed, we posted the Tele-OCS pack at least 1 week before the appointment and reminded them not to open the pack until instructed. In the session, informed consent was taken and the Tele-OCS was administered either by phone or videoconferencing depending on the preference of the participant. Shifting modality during a session was not permitted, nor did this come up during data collection. We provided a pre-paid envelope for participants to post their packs back. If participants were recruited from the OXCHRONIC study, we contacted them within 30 days of having completed the Tele-OCS for the OXCHRONIC protocol, and administered the OCS in person at their homes.

### Data analysis

Prior to formal analyses we examined sample characteristics, including demographics and prior OCS performance taken within 20 days of stroke. Acute OCS performance is presented in Table S2 in the extended data.

First, we summarized the administration time of participants and comments given by participants about the Tele-OCS. Cut offs for impairment per OCS subtask were created using the original OCS normative cut offs from healthy aging data ( Demeyere *et al.*, 2015). Then we visually depicted proportion of impaired subtasks per modality and impairment classification per sub-task for both modalities. We further explored continuous performance scores of participants who were classed as unimpaired on the in-person OCS but impaired on the Tele-OCS, and vice versa. We compared raw continuous performance scores for statistically significant differences uses *t*-tests and Cohen’s *d* effect size.

All statistical analysis and data sorting was computed in R Studio ( Posit team, 2024) using R ( R Core Team, 2021) version 4.0.4. We used the following packages for the production of the RMarkdown manuscript and analysis: *bookdown* version 0.26 ( Xie, 2021); *readxl* version 1.3.1 ( Wickham & Bryan, 2019); *cowplot* version 1.1.1 ( Wilke, 2020); *ggplot2* version 3.3.5 ( Wickham, 2016); *kableExtra* version 1.3.4 ( Zhu, 2021); *httr* version 1.4.2 ( Wickham, 2022); and *tidyr* 1.2.0 ( Wickham, 2021). Data and analysis scripts to recreate the manuscript are openly available in CC-BY 4.0 license ( Webb *et al.*, 2023).

## Results

### Sample

40 chronic stroke survivors took part in this study, with demographics of participants presented in Table 1. The median time post-stroke was 228 days ( *IQR=189.25 to 1500.50).* One participant did not return their Tele-OCS pack, as such we were unable to generate scores for the number writing, broken hearts, or trails accuracies subtasks. We include this participant in the majority analyses where complete data were available, including proportion of subtasks and domains impaired as proportion accounts for missing subtasks. We present OCS data taken within 20 days of stroke in the extended data for context on generalisability of the current sample and impairment levels.

**Table 1.** Demographics of the participants included in the final analysis.

Characteristic	N (missing%)	Value
Age (M ( *SD*))	40 (0%)	69.30 (10.44)
Education (M ( *SD*))	40 (0%)	13.45 (2.85)
Sex	40 (0%)	Female: 30%; Male: 70%;
Ethnicity	40 (0%)	Black-Caribbean: 2.5%; White-British: 97.5%;
Stroke type	40 (0%)	Subarachnoid haemorrhage: 2.5%; Intracerebral haemorrhage: 27.5%; Ischaemic: 70%;
Stroke side	40 (0%)	Bilateral: 10%; Left: 45%; Right: 45%;
NIHSS (median (IQR))	37 (8%)	7 (3–9)

Note. NIHSS refers to National Institute of Health Stroke Scale for stroke severity. IQR refers to interquartile range.

### Tele-OCS overview

The time in days between in-person and Tele-OCS administration ranged from 6 to 72, with an average of *M*=27.70 days ( *SD*=12.29). 23 participants were administered the Tele-OCS first (57.5%). Time interval was not correlated with any differences in subtasks impaired on either modality ( *r*(38)= -0.03, *p*=.85). Administration of the Tele-OCS took an average time of 17.17 minutes ( *SD*=2.73, range = 14 - 22). The Tele-OCS was administered using phone to the majority of participants ( *n*=36, 90%).

14 participants volunteered feedback after the Tele-OCS was administered. Nine participants commented that there were limited differences in their experience between in-person and remote administration. Three participants had issues with their phone connection, which broke up the session slightly, but minimally impacted administration. One person had difficulty finding the correct pages and, due to a hemiplegia, required help from his partner to turn the pages. One participant required a repetition of one instruction once due to hearing difficulties. Otherwise, participant remote sessions were administered without major barriers.

### Comparison of the Tele-OCS and standard OCS

**Figure 1.**
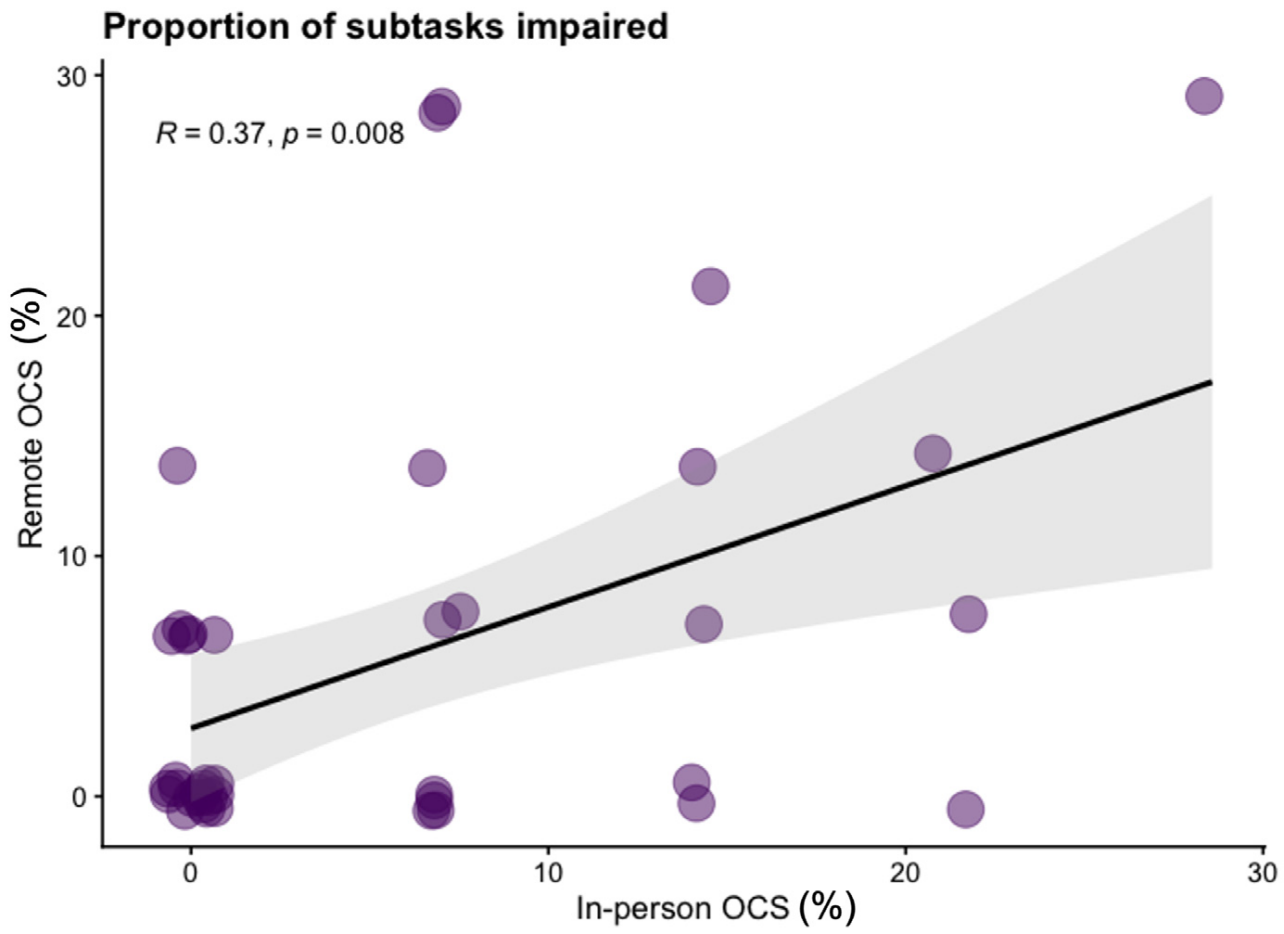
Scatter plot of the proportion of subtasks completed which are classified as impaired on the in-person Oxford Cognitive Screen (OCS) and Tele-OCS. R = Kendall rank correlation coefficient. Figure available under CC-BY 4.0 license (Figure 1, Webb *et al.*, 2023).

**Figure 2.**
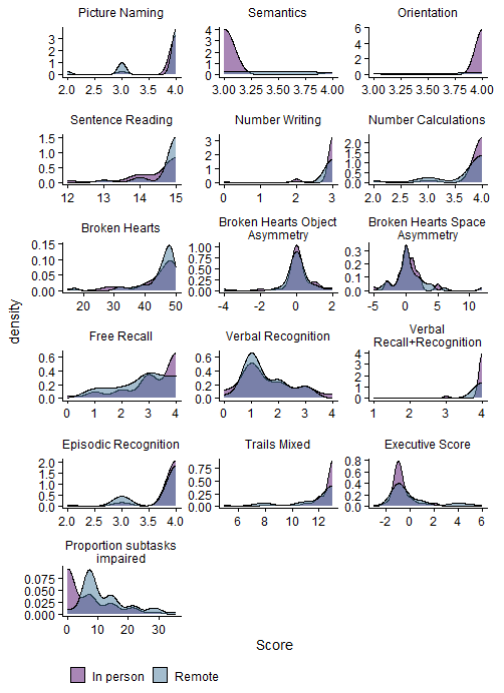
Illustrates the density of scores per subtask, and per OCS modality (coloured by in-person Oxford Cognitive Screen (OCS) or Tele-OCS). Figure available under CC-BY 4.0 license (Figure 2, Webb *et al.*, 2023).

We examined the proportion of subtasks impaired on the in-person versus Tele-OCS, and then the frequency of impairment classifications for each OCS subtask across modalities. Participants were impaired on an average of 5.71 ( *SD*=7.79) subtasks on the in-person version and 5.71 ( *SD*=8.59) subtasks in the remote version. In Figure 1 we present the Kendall rank correlation coefficient for proportion of subtasks impaired between remote and in-person OCS versions. We visually depict impairment classification distributions for all OCS scores in Figure 2 to illustrate the similarities / differences between modalities. Figure 2 shows that, in terms of count, there were a few individuals who were impaired only on the remote version of the task, but not in the in-person version. In Table 2, we present descriptive statistics for raw score performance on each shared OCS subtask as well as repeated-measures Cohen’s *d* and significance of difference on *t*-tests between each subtask. Equivalence tests were additionally conducted, paralleling the t-test results presented here (see Supplementary Materials).

**Table 2.** Descriptive statistics for subtasks per domain on the Tele-OCS and in-person OCS.

Domain	Subtask	N	In-person M(SD)	Remote M(SD)	Cohen’s *d*
Language	Picture Naming	40	3.92 (0.27)	3.70 (0.56)	0.36 *
	Semantics	40	3.05 (0.22)	3 (0)	0.23
	Sentence Reading	40	14.53 (0.82)	14.80 (0.52)	-0.31
Number	Number Writing	39	2.92 (0.27)	2.87 (0.52)	0.11
	Number Calculations	40	3.90 (0.38)	3.75 (0.54)	0.23
Memory	Orientation	40	3.98 (0.16)	4 (0)	-0.16
	Verbal Recall Recognition	40	3.95 (0.22)	3.83 (0.59)	0.24
	Episodic Recognition	40	3.88 (0.4)	3.80 (0.41)	0.13
Attention	Broken Hearts	39	43.85 (7.96)	44.67 (6.41)	-0.20
	Broken Hearts Object Asymmetry	39	0.17 (0.68)	0 (0.83)	0.24
	Broken Hearts Space Asymmetry	39	0.50 (2.65)	0.67 (2.74)	-0.05
Executive	Trails Mixed	39	11.03 (2.88)	11.74 (2.11)	0.26
	Executive Score	39	-0.68 (0.86)	0.10 (2.02)	-0.46 *
Overall	Subtasks Impaired (%)	40	5.71 (7.79)	5.71 (8.59)	0

Note. ‘*’ signifies *p*-value <.05. Multiple comparison alpha corrected level is .005 (.05/11 = .005). ‘**’ signifies *p*-values <.005).

The results show that regardless of numerical differences in score, there were few statistically significant differences between raw performance scores on each subtask and overall proportion of tasks impaired. We present sensitivity/specificity analysis in the extended data as not all analysis could be completed due to low and differing numbers of impaired impairment classifications.

## Discussion

We aimed to validate the use of the Tele-Oxford Cognitive Screen (Tele-OCS) by comparing performance within-subjects on the in-person OCS (OCS version B) and remote OCS (Tele-OCS version A), via telephone or video conferencing. Overall, the proportion of those impaired on the OCS subtasks was moderately related between in-person and remote administration. Within each of the subtasks, whilst we found small statistically significant deviations in raw scores in two tasks (picture naming and executive score), impairment classifications on each task were minimally affected by modality.

It is possible that differences in OCS versions (A vs B) led to increased variability in performance on OCS subtasks between modalities. This may be particularly the case for the picture naming task, where two participants were impaired on the Tele-OCS (based on version A), but not on the in-person OCS (based on version B). There are small differences between version A and B of the picture-naming task, irrespective of modality. For example, participants are more likely to make errors on version A, by calling the hippo a ‘rhino’ and calling the slices of melon, ‘fruit’ (see OCS control normative data, Demeyere *et al.*, 2015; in Webb *et al.*, 2023). For the executive score, although scores between modalities were statistically different, impairment ranges were nearly identical, with only one participant classed as impaired on the remote version and unimpaired on the in-person version.

Our results align with other validation studies of remote versions of commonly used cognitive screening tools. One longitudinal study comparing performance on telephone versus in-person administration of a cognitive test battery of multiple domains (attention, verbal learning and memory, verbal fluency, executive function, working memory and global cognitive functioning) ( Rapp *et al*., 2012). Statistical differences were found between in-person and remote assessment scores, with the most reliable test-retest data found using the same modality ( Rapp *et al.*, 2012). Despite these small differences, remote and in-person administrations yielded equivalent interpretations in most cases, with the authors concluding that this remotely assessed battery was comparable to its in-person version. Katz *et al.* (2021) compared performance on the MoCA and Tele-MoCA, finding small but non-meaningful differences in performance between modalities (e.g., non-significant equivalence tests). Overall, prior research, along with the results of the present investigation, suggest that slight differences between remote and in-person versions of the same cognitive test are to be expected. It is possible that continuous performance scores, when delivered remotely, may be more likely to fluctuate, possibly due to technical problems, lack of examiner presence to guide correct instructions (e.g., for the trail-making task), and hearing or language difficulties that become exacerbated during remote cognitive assessment. However, overall differences are minor, and should not be a barrier to administering cognitive tests in remote format nor to overall interpretation of participant results. The Tele-OCS thus provides a highly comparable assessment of cognitive functioning.

There are several stroke-specific considerations concerning remote cognitive assessments. For instance, motor impairments may affect stroke survivors’ ability to complete cognitive tests in remote format. One participant in the present study remarked that turning pages in the Tele-OCS booklet was difficult due to their hemiplegia. Motor difficulties such as these should be taken into consideration by clinicians when assessing stroke survivors remotely, so that best-practice may be established. Additionally, visuospatial neglect, a common post-stroke impairment, may impact remote cognitive test performance. Participants should be reminded to keep their remote testing booklet as close to their mid-line as possible so that cognitive performance in other domains is not impacted by neglect symptoms. The in-person and Tele-OCS are designed to be spatial neglect friendly, with stimuli presented centrally on all pages. Involvement of a family member or carer at home when conducting a remote assessment may be beneficial for participants that require additional support where in person assessment is not possible.

Remote cognitive assessment has multiple benefits. Delivering cognitive assessments in remote format can increase the accessibility of these assessments to a wider range of individuals ( Webb *et al.*, 2022). Similarly, remote cognitive assessments can reduce participant burden for those participating in research studies, given that individuals do not have to be physically present to participate. Those who would not usually participate in research may be encouraged to do so if the modality of assessment is remote. It should be noted that the present investigation was the first to partially validate a *stroke-specific* cognitive screening tool for remote use. Given that commonly used remote assessments, such as the MoCA and Tele-MoCA, do not usually assess stroke-specific cognitive impairments (e.g., unilateral spatial neglect and aphasia; Laska *et al.*, 2001; Moore *et al.*, 2021), the Tele-OCS can improve the accessibility of adequate cognitive screening to stroke survivors during their recovery, increasing the likelihood of appropriate cognitive review if necessary.

### Limitations

Although our study sample was statistically powered, the sample size was small and relatively homogeneous. For example, 97.5% of the sample was White-British, 70% were male, and around 70% were ischaemic stroke. Ideally, there would be around 82% White-British participants to reflect UK government 2021 census data for ethnicity ( ONS, 2022), or >93% for older adults specifically, or 87% for general UK stroke survivors ( Douiri *et al.*, 2021), approximate equality of males and female, and around 87–90% ischemic stroke ( Douiri *et al.*, 2021; King *et al.*, 2020). Strengths of our sample, however, include age and stroke severity, which represent the general UK stroke population ( Douiri *et al.*, 2021).

Furthermore, given that many participants were in the chronic stage of stroke, they were often classified as unimpaired across subtasks in both modalities, limiting our interpretation for stroke survivors with more severe impairments. This may impact the generalisability of the present results to the wider stroke population. Although the majority of stroke survivors tend to be males, our weighting of males to females was biased as we had far more males. This may impact the generalisability of our results to females. We do not feel that the gender balance in our data impacts the results drastically, however, we acknowledge this as a limitation. We did not choose to purposively sample for sex specifically as there is limited and mixed evidence that sex alters global cognitive scoring after a stroke, however, sex differences do exist in pre-stroke risk factors, as well as type of stroke and non-cognitive recovery which we did not factor in this study ( Duran *et al*., 2024; Indraswari *et al*., 2024). Future validation studies of the Tele-OCS may benefit from using larger, more heterogeneous samples, including those who have sustained more severe strokes, and more representative demographics. Moreover, it is necessary to consider that those who consent to take part in research may be different from those who do not, and as such more inclusive study protocols using those with in-capacity to consent to research may help contribute to more reflective demographics. There are reported differences in demographics between those who have capacity to consent to take part in a research study versus those who do not; typically those without capacity in this context are older, more neurologically impaired, and more frequently have aphasia ( Lindley *et al*., 2022; Thomalla *et al*., 2017). 

There is currently no normative data for performance on the Tele-OCS for comparison of scores, as our results rely on the in-person normative data to generate impairment classifications we cannot know if these impairment classifications are accurate. Further research will need to determine this. Furthermore, a small part of our study concerned different modalities of remote assessment (phone vs videocall). We did not aim to assess whether the modality of remote assessment affected performance. We relied on participant preference above all else to determine modality.

Additionally, we had too small a sample to do so. Given identical instructions and materials, there should be limited differences between modalities. Furthermore, it should be noted that stroke survivors, and indeed current older adults, do not always have the correct equipment or support to carry out assessment via videoconferencing. There are multiple barriers for older adults including patient acceptability, software used to make the call, hardware to do so, and health impairments related to independently hosting a videoconference and completing tasks simultaneously ( Niyomyart *et al*., 2024). Additionally, while internet access is common for stroke survivors, the ones with more access tend to be younger, white, and live longer, introducing further biases to research samples ( Naqvi *et al*., 2021; Zhu *et al*., 2022). Indeed, other equivalence/videoconferencing studies have used younger, less impaired samples than the current study, potentially explaining their high use of videoconferencing and assumed practicality for older aged stroke survivors ( Chapman *et al*., 2021; Gnassounou *et al*., 2022). However, it may be worthwhile for future studies with larger sample sizes to randomly allocate participants to either telephone or videoconferencing to investigate feasibility in older aged stroke survivors with cognitive impairment whether there are any differences between these types of remote assessment methods.

Finally, all participants were previously exposed to the OCS, whether as part of our research, or as part of their admission to hospital where OCS is given as standard where it can be. This means, there may be a chance for practice effects. We note, we did not observe many in the current study, so it is reasonable to assume less exist between first exposure and the current study. Alternatively, due to previous exposure, participants may be better at the OCS in general, which would explain our low rates of impairment. As other studies using the OCS have found impairment rates, even for those previously exposed, and at different time points post-stroke ( Kusec *et al.*, 2023; Milosevich *et al.*, 2023), we do not feel this is a major issue in the current study.

### Implications for research and clinical practice

The present investigation provides insight into how to best administer the Tele-OCS. Sending a paper pack in the post allows for clear presentation of instructions and stimuli while carrying out Tele-OCS assessment. However, there are also several drawbacks to this method; participants must be instructed not to look at the testing pack ahead of the testing session and it can be time-consuming (and potentially costly to NHS services) to send the pack via post. However, use of a paper testing pack allows stroke survivors who do not have access to videoconferencing to carry out the testing session over the telephone, without having to travel to a research or clinic site. In our sample, the majority of participants opted to complete the Tele-OCS over the phone, rather than using videoconferencing. This may be because they are less familiar with, or do not have access to, the required technology.

We encourage health professionals to take these factors into consideration when choosing to use the Tele-OCS. Clinicians who are new to remote assessment are encouraged to carry out practice administrations with the Tele-OCS before using it in a clinical context.

## Conclusion

The OCS, when administered remotely, is a valid method of screening for cognitive impairment among stroke survivors. Clinicians should use existing published normative data to interpret impairment on OCS sub-tests when delivered remotely.

## Data Availability

Open Science Framework: Introducing the Tele-OCS: A validated remotely administered version of The Oxford Cognitive Screen. https://doi.org/10.17605/OSF.IO/8RNY2 ( Webb *et al.*, 2023) This project contains the ‘Reproducible manuscript’ folder which you can download as a .zip file, and using the .Rproj file, can reproduce our base manuscript and analyses. This also contains ‘data’ subfolder with this research’s underlying data. Open Science Framework: Introducing the Tele-OCS: A validated remotely administered version of The Oxford Cognitive Screen. https://doi.org/10.17605/OSF.IO/8RNY2 ( Webb *et al.*, 2023) This project contains the ‘Supplementary materials’ folder. Data are available under the terms of the Creative Commons Attribution 4.0 International license (CC-BY 4.0).
