# Supplementary material for: Introducing the Tele-OCS: Preliminary evidence of validity for a remotely administered version of The Oxford Cognitive Screen
Source: Health Open Res. 2025 Jul 9;5:8. Originally published 2023 Feb 13. [Version 3] doi: 10.12688/healthopenres.13291.3 (PMC12287908; doi:10.12688/healthopenres.13291.3)
Supplement: Peer review history

# Reviewer report: Reviewer response for version 3

**DOI:** 10.21956/healthopenres.15001.r29461

**Published:** 2025-09-10

**Authors:** Hayley Wright

**Affiliations:** 1 Coventry University, Coventry, England, UK

Thank you for your careful consideration of my comments and suggestions. This articles adds a significant contribution to knowledge on remotely administered cognitive screening. I would be delighted to see this indexed in it's current form.

Is the work clearly and accurately presented and does it cite the current literature?

Partly

If applicable, is the statistical analysis and its interpretation appropriate?

Yes

Are all the source data underlying the results available to ensure full reproducibility?

Yes

Is the study design appropriate and is the work technically sound?

Yes

Are the conclusions drawn adequately supported by the results?

Yes

Are sufficient details of methods and analysis provided to allow replication by others?

Yes

Reviewer Expertise:

Cognitive psychology; digital health interventions

I confirm that I have read this submission and believe that I have an appropriate level of expertise to confirm that it is of an acceptable scientific standard.

# Reviewer report: Reviewer response for version 3

**DOI:** 10.21956/healthopenres.15001.r29462

**Published:** 2025-08-01

**Authors:** Léon Franzen, Frederike Schröpfer

**Affiliations:** 1 Department of Psychiatry and Psychotherapy, University of Lübeck, Lübeck, Schleswig-Holstein, Germany

Thank you for uploading the supporting materials to the OSF repository and addressing my comments. I have no further comments to make.

Is the work clearly and accurately presented and does it cite the current literature?

Yes

If applicable, is the statistical analysis and its interpretation appropriate?

Partly

Are all the source data underlying the results available to ensure full reproducibility?

Yes

Is the study design appropriate and is the work technically sound?

Partly

Are the conclusions drawn adequately supported by the results?

No

Are sufficient details of methods and analysis provided to allow replication by others?

Yes

Reviewer Expertise:

Cognitive neuroscience and psychology, psychiatry, remote assessments

We confirm that we have read this submission and believe that we have an appropriate level of expertise to confirm that it is of an acceptable scientific standard.

# Reviewer report: Reviewer response for version 2

**DOI:** 10.21956/healthopenres.14761.r29448

**Published:** 2025-07-07

**Authors:** Hayley Wright

**Affiliations:** 1 Coventry University, Coventry, England, UK

Thank you for the opportunity to review this paper. This is a novel and strong contribution to an under-researched and under-resourced area of stroke care. Overall, the paper is excellent and gives a thorough account of the validity-testing of remote vs in-person testing of a stroke-specific and domain-specific cognitive screen. 

 Minor comment for the introduction (first paragraph) is to cite more recently updated stroke guidelines, such as the National Clinical Guideline for Stroke (2023).

 Methods: "In the current study we attempted to reflect the UK stroke population in key demographics of age, education, sex, stroke type, stroke side, and stroke severity." Can the authors please clarify how this was done? Perhaps stratified sampling or similar? Can they also clarify how this reconciles with the following statement further down "Recruitment was conducted irrespective of participant demographics..."

 Can the authors add some justification for why the Tele-OCS was based on Version A of the OCS (rather than version B, or creating a remote version for both)?

 By recruiting participants from the OCS programme and OXCHRONIC, are you introducing any test-retest bias to the results? That is, do practice effects improve performance, such that any differences between modalities are minimised by exposure, rather than the method of administration per se? This is mentioned in the limitations section in the discussion but does not satisfy my doubts as a reader. Can further evidence be presented to support the limited practice effects from other studies?

 The limitations section of the discussion is thoughtful but needs supporting with references. In paces, the suggestions feel anecdotal or vague. For example:

-  "We did not choose to purposively sample for sex specifically as it is not thought to alter the results of global cognitive scoring after a stroke" - reference needed. 
- "Moreover, it is necessary to consider that those who consent to take part in research may be different from those who do not, and as such more inclusive study protocols using those with in-capacity to consent to research may help contribute to more reflective demographics." - reference to support that ways in which those who lack capacity to consent may hold different demographic characteristics to those who have capacity.
- "Furthermore, it should be noted that stroke survivors do not always have the correct equipment to carry out assessment via videoconferencing" - reference? This feels like a sweeping statement - could potentially be perceived as stereotyped / prejudiced in its tone. Can you be clear about whether this was borne out in your data, or where the evidence comes from to support this, and how it can be remedied in future research? E.g. providing / loaning smartphones or tablets for participants to complete remote assessments with.

Minor note about grammar - some sentences are very long and hard to follow, e.g. "We did not aim to assess whether the modality of remote assessment affected performance, as we relied on participant preference above all else, had too small a sample to do so, given identical instructions and materials, there should be limited differences". Breaking down into shorter sentences will help readers (particularly lay, or less-strong, readers) to understand the importance and impact of the work.

Is the work clearly and accurately presented and does it cite the current literature?

Partly

If applicable, is the statistical analysis and its interpretation appropriate?

Yes

Are all the source data underlying the results available to ensure full reproducibility?

Yes

Is the study design appropriate and is the work technically sound?

Yes

Are the conclusions drawn adequately supported by the results?

Yes

Are sufficient details of methods and analysis provided to allow replication by others?

Yes

Reviewer Expertise:

Cognitive psychology; digital health interventions

I confirm that I have read this submission and believe that I have an appropriate level of expertise to confirm that it is of an acceptable scientific standard, however I have significant reservations, as outlined above.

# Response

**Published:** 2025-07-07

**Authors:** Sam Webb

**Affiliations:** University of Oxford, UK

**Response to reviewer** We thank the reviewer for providing feedback on this manuscript draft. Please find below a detailed response to each comment.   Reviewer comments are in italics, our reply is in bullet point format in plain text, and all text extracted from the manuscript are in quotations with changes in red. **Reviewer 3:** **Minor comment for the introduction (first paragraph) is to cite more recently updated stroke guidelines, such as the National Clinical Guideline for Stroke (2023).**  

- We have now added in the more current clinical guidelines at the end of the first paragraph.

 
**Methods: "In the current study we attempted to reflect the UK stroke population in key demographics of age, education, sex, stroke type, stroke side, and stroke severity." Can the authors please clarify how this was done? Perhaps stratified sampling or similar? Can they also clarify how this reconciles with the following statement further down "Recruitment was conducted irrespective of participant demographics..."**

- We have removed this to lessen confusion, as we did not have formal methods for doing checking bias of demographics in the sample.  

   
**Can the authors add some justification for why the Tele-OCS was based on Version A of the OCS (rather than version B, or creating a remote version for both)?**

- Thank you for raising this. We have now addressed this in the Tele-OCS section:
- “The remote version of the OCS is an adaptation of version A of the OCS. Version A is currently the only version used here, as this was the form used in OxCHRONIC which did not repeat testing with version B. A version B will be available in the future, but is not used in the current study. Administration of the Tele-OCS can be conducted via telephone or video-call.”

   
**By recruiting participants from the OCS programme and OXCHRONIC, are you introducing any test-retest bias to the results? That is, do practice effects improve performance, such that any differences between modalities are minimised by exposure, rather than the method of administration per se? This is mentioned in the limitations section in the discussion but does not satisfy my doubts as a reader. Can further evidence be presented to support the limited practice effects from other studies?**

- Thank you for the comment and highlighting an important flaw in many equivalence studies. We have controlled for this somewhat we counterbalancing within the sample so that some get in-person OCS first, then the Tele-OCS. We have explicitly stated this in the Study design and sample size section:
- “This means the sampling approaches originally used have minimal bias (except bias of those who are willing to participate), and the underlying samples are representative of the UK stroke population. We acknowledge that by study design, participants will be exposed to the same test twice and no matter the order of administration (remote or in-person) they may improve on the second test. The study design is typical and is shared with multiple other comparison studies of modality equivalence (Brown et al., 2024; Duran et al., 2024; Morrissey et al., 2024). We used two methods to address possible practice effects: We ensured that different sets of the sample receive opposite administration order (Tele-OCS vs. OCS) and different versions (A or b). We further used group statistics to examine if participants improve on their second administered test.”

   
**The limitations section of the discussion is thoughtful but needs supporting with references. In paces, the suggestions feel anecdotal or vague. For example:** **"We did not choose to purposively sample for sex specifically as it is not thought to alter the results of global cognitive scoring after a stroke" - reference needed. **

- We than you for highlighting the non-scientific tone, we agree. We have now rephrased and included citations.
- **“**We do not feel that the gender balance in our data impacts the results drastically, however, we acknowledge this as a limitation. We did not choose to purposively sample for sex specifically as there is limited and mixed evidence that sex alters global cognitive scoring after a stroke, however, sex differences do exist in pre-stroke risk factors, as well as type of stroke and non-cognitive recovery which we did not factor in this study (Duran et al., 2024; Indraswari et al., 2024).”

   
**"Moreover, it is necessary to consider that those who consent to take part in research may be different from those who do not, and as such more inclusive study protocols using those with in-capacity to consent to research may help contribute to more reflective demographics." - reference to support that ways in which those who lack capacity to consent may hold different demographic characteristics to those who have capacity.**

- We have now added this in and adjusted test:
- “Moreover, it is necessary to consider that those who consent to take part in research may be different from those who do not, and as such more inclusive study protocols using those with in-capacity to consent to research may help contribute to more reflective demographics. There are reported differences in demographics between those who have capacity to consent to take part in a research study versus those who do not; typically those without capacity in this context are older, more neurologically impaired, and more frequently have aphasia (Lindley et al., 2022; Thomalla et al., 2017).”

     
**"Furthermore, it should be noted that stroke survivors do not always have the correct equipment to carry out assessment via videoconferencing" - reference? This feels like a sweeping statement - could potentially be perceived as stereotyped / prejudiced in its tone. Can you be clear about whether this was borne out in your data, or where the evidence comes from to support this, and how it can be remedied in future research? E.g. providing / loaning smartphones or tablets for participants to complete remote assessments with.**

- Thank you for your comment here. We did not wish to phrase our discussion in such a way to bring in prejudice. We have significantly altered this section to providence evidence for our claims.
- “Furthermore, it should be noted that stroke survivors, and indeed current older adults, do not always have the correct equipment or support to carry out assessment via videoconferencing. There are multiple barriers for older adults including patient acceptability, software used to make the call, hardware to do so, and health impairments related to independently hosting a videoconference and completing tasks simultaneously (Niyomyart et al., 2024). Additionally, while internet access is common for stroke survivors, the ones with more access tend to be younger, white, and live longer, introducing further biases to research samples (Naqvi et al., 2021; Zhu et al., 2022). Indeed, other equivalence/videoconferencing studies have used younger, less impaired samples than the current study, potentially explaining their high use of videoconferencing and assumed practicality for older aged stroke survivors (Chapman et al., 2021; Gnassounou et al., 2022). However, it may be worthwhile for future studies with larger sample sizes to randomly allocate participants to either telephone or videoconferencing to investigate feasibility in older aged stroke survivors with cognitive impairment and whether there are any differences between these types of remote assessment methods.”

     
**Minor note about grammar - some sentences are very long and hard to follow, e.g. "We did not aim to assess whether the modality of remote assessment affected performance, as we relied on participant preference above all else, had too small a sample to do so, given identical instructions and materials, there should be limited differences". Breaking down into shorter sentences will help readers (particularly lay, or less-strong, readers) to understand the importance and impact of the work.**

- Thank you for highlighting this to us. We have inspected the whole article and broken down long sentences for clearer understanding.

# Reviewer report: Reviewer response for version 2

**DOI:** 10.21956/healthopenres.14761.r28061

**Published:** 2024-04-11

**Authors:** Dimosthenis Tsapekos

**Affiliations:** 1 King's College London, London, England, UK

The article has been much improved.

Is the work clearly and accurately presented and does it cite the current literature?

Yes

If applicable, is the statistical analysis and its interpretation appropriate?

Yes

Are all the source data underlying the results available to ensure full reproducibility?

Yes

Is the study design appropriate and is the work technically sound?

Yes

Are the conclusions drawn adequately supported by the results?

Yes

Are sufficient details of methods and analysis provided to allow replication by others?

Partly

Reviewer Expertise:

Neuropsychology

I confirm that I have read this submission and believe that I have an appropriate level of expertise to confirm that it is of an acceptable scientific standard.

# Reviewer report: Reviewer response for version 1

**DOI:** 10.21956/healthopenres.14374.r27335

**Published:** 2023-08-17

**Authors:** Dimosthenis Tsapekos

**Affiliations:** 1 King's College London, London, England, UK

- In the abstract a value of d=0 is reported, is this a typo? It's quite unusual for an effect size metric to be exactly 0. 
- The introduction sufficiently motivates the need to validate a remotely administrated version of the OCS and the conduct of the study. 
- The issue of previous participation in research needs to be clarified. Does this involve previous administration of the OCS? This could affect the outcomes through learning effects.
- The two versions of the measure are sufficiently described. 
- Data analysis description is complete.
- Visual presentation of data facilitates the understanding of the findings.
- The authors present impairment rates for subtasks. It's not clear whether there is impairment classification based on the overall score of the two OCS versions. This would be very useful to clarify and report. 
- Limitations/Implications are sufficiently described. 
- I think it's important for the authors to delineate the previous experience participants had with the two OCS measures. This was the only confusing part.  

Is the work clearly and accurately presented and does it cite the current literature?

Yes

If applicable, is the statistical analysis and its interpretation appropriate?

Yes

Are all the source data underlying the results available to ensure full reproducibility?

Yes

Is the study design appropriate and is the work technically sound?

Yes

Are the conclusions drawn adequately supported by the results?

Yes

Are sufficient details of methods and analysis provided to allow replication by others?

Partly

Reviewer Expertise:

Neuropsychology

I confirm that I have read this submission and believe that I have an appropriate level of expertise to confirm that it is of an acceptable scientific standard, however I have significant reservations, as outlined above.

# Response

**Published:** 2024-03-15

**Authors:** Sam Webb

**Affiliations:** University of Oxford, UK

Title: ‘Introducing the Tele-OCS: Preliminary evidence for a remotely administered version of The Oxford Cognitive Screen’  

**Response to reviewers** We thank the reviewers and the editors for providing feedback on this manuscript draft. Please find below a detailed response to each reviewer comment.   Reviewer comments are in italics, our reply is in bullet point format in plain text, and all changes made in the manuscript text are in red.  

**1. *In the abstract a value of d=0 is reported, is this a typo? It's quite unusual for an effect size metric to be exactly 0. ***

-
  - *The* *d *= 0 effect size was due to rounding the value to 2 decimal places. We have now adjusted this in the abstract to be more precise, as below:

--- We found that the proportion of OCS subtasks impaired did not differ across modalities ( *d* = <.001). ---  

***2.      The introduction sufficiently motivates the need to validate a remotely administrated version of the OCS and the conduct of the study. ***

- We thank the reviewer for their kind words.

**3.******* The issue of previous participation in research needs to be clarified. Does this involve previous administration of the OCS? This could affect the outcomes through learning effects. I think it's important for the authors to delineate the previous experience participants had with* **the two OCS measures. This was the only confusing part.  **

- Reviewer 1 has also addressed this point (see comment 1), We have now clarified that all participants, and most stroke survivors in Oxfordshire, have attempted the OCS at least once while in acute care therefore practice effects are to some degree unavoidable. We have also addressed this in our limitations section:

  --- Finally, all participants were previously exposed to the OCS, whether as part of our research, or as part of their admission to hospital where OCS is given as standard where it can be. This means, there may be a chance for practice effects. We note, we did not observe many in the current study, so it is reasonable to assume less exist between first exposure and the current study. Alternatively, due to previous exposure, participants may be better at the OCS in general, which would explain our low rates of impairment. As other studies using the OCS have found impairment rates, even for those previously exposed, and at different time points post-stroke (Kusec *et al*., 2023; Milosevich *et al*., 2023), we do not feel this is a major issue in the current study. ---  

**4.******* The two versions of the measure are sufficiently described.* ***Data analysis description is complete.*** ***Visual presentation of data facilitates the understanding of the findings.*** ***Limitations/Implications are sufficiently described. ***

- We thank the reviewer for their kind comments on our methods, analyses, figures, and limitations section.

 
**5. *****The authors present impairment rates for subtasks. It's not clear whether there is impairment classification based on the overall score of the two OCS versions. This would be very useful to clarify and report. ***

- We thank the reviewer for highlighting this for clarification, we hope we have now clarified which tasks we are referring to; see below:

--- We examined the proportion of subtasks impaired on the in-person versus Tele-OCS, and then the frequency of impairment classifications for each OCS subtask across modalities. Participants were impaired on an average of 5.71 ( *SD*=7.79) subtasks on the in-person OCS and 5.71 ( *SD*=8.59) subtasks in the Tele-OCS. ---

# Reviewer report: Reviewer response for version 1

**DOI:** 10.21956/healthopenres.14374.r27286

**Published:** 2023-08-04

**Authors:** Léon Franzen, Frederike Schröpfer

**Affiliations:** 1 Department of Psychiatry and Psychotherapy, University of Lübeck, Lübeck, Schleswig-Holstein, Germany

Webb et al. attempt to provide an important development of the OCS by attempting to validate the remote version of the Oxford Screening Tool administered via telephone or videocall. The Covid-19 pandemic has shown us that many studies and assessments can also be delivered online providing somewhat comparable results. The benefits of remote assessments and studies are undisputed. However, remote delivery comes with considerable challenges including much reduced control over the test administration and testing environment. This potential pitfall is exacerbated when delivering an assessment via telephone to potentially cognitively impaired patients. In the present manuscript, by postulating similar impairment classification of the same participants even under changed conditions, Webb et. al. test their remote OCS version and conclude it to be a valid version across telephone and videocall delivery channels. While I appreciate their open science practices, particularly their sampling and testing practices come with a number of considerable shortfalls that do not allow for drawing the all-encompassing conclusion of the remote OCS being a widely validated test after this rather small sample study. I will elaborate on and discuss these points in more detail below.

**Major Issues**

***Recruitment and sample characteristics***

 With their study to validate the remotely administered oxford cognitive screen (OCS), Webb et al. attempted a previously analogue assessment of cognitive performance. One crucial issue of the study lies in the recruitment of participants. The authors used the strategy of community dwelling to recruit their participants but these individuals were sourced from completed studies of the Oxford Cognitive Screening Programme or other studies which tested or trained the cognitive performance of patients after a stroke. In my opinion, this creates a recruitment bias. Due to the prior participation in other studies, whereby the selection of participants is not at random and thus the sample is not representative of the underlying population. As well, since patients were recruited as part of a 6 month follow up and also after other studies, it would be important to clarify whether these participants had already done the OCS at least once within the first months after their stroke, as I presume the potential for practice effects exists. However, this piece of information is not provided. This could be tested by comparing reliability between participants who had taken the test before starting this study and those who have not.

 Similarly, the existing study design results in an increased risk of selection bias, as only participants who completed the previous studies were included. If one recruits only from the group of test persons who have already completed a study, one may suspect that these are particularly motivated and performance-oriented individuals. Hence, generalisability to the general population may not be given.

 As previously mentioned, the Information regarding the participants is rather limited. Webb et al. reported most participants being white males as a weakness of the study. They did also not address the presence of ischemic strokes either, which account for 70% of their sample. This is much lower than the prevalence reported in previous studies of around 87% (Busch & Kuhnert, 2017; Saini et al., 2021), which may affect generalisability. It may also come with different symptoms compared to the presence of subarachnoid and intracerebral haemorrhages. A more precise, detailed presentation of the included participants would be desirable for the reader.

 Following on from this point, generalisability to the entire British population or beyond and to various remote delivery channels is also questionable, given the small sample (N=40), the heavy reliance on white British males and most of these participants taking the test on the telephone. To warrant the authors claims of presenting a successfully validated test, the sample size would need to be increased and sample’s characteristics more balanced.

 Further, the authors do not address the interval between the stroke and the time of the assessment. Here, too, a more detailed description and analysis would be desirable due to the following reasons.

 The time post-stroke in which participants took part varied largely. The IQR suggests that some had a twice as long (or an even longer) recovery period before they participated in the study. Some participants were included after more than a 6.5 times longer recovery time (1500/228 days) compared to the median. Since the study design was within-subjects this aspect may not matter too much by itself but contributes to much heterogeneity in recovery possibilities within an already small sample.

 Following on from this, the within-subject test-retest interval between the in-person and remote tests shows some considerable range as well. Here, I’m doubtful that a gap of 6 days is comparable with one of 72 days. Hence, I’d suggest to the authors to ensure that participants with a shorter interval did not show more consistency in their results compared to those with an interval longer than the mean. This could also be tested using a correlative approach correlating test-retest interval and the difference in % of the proportion of subtasks impaired.

 The inclusion/exclusion criteria of the study were described by Webb et al. but appear to miss some important information. On the one hand, participants have a history of a confirmed stroke, but the severity or location of the stroke is not described. May this have an effect? I would also like to see the authors provide a more detailed description of the included participants and how their characteristics relate to the results, as no information on relevant symptoms such as neglect or hemiparesis among the participants other than may be indirectly obtained from the broken hearts subtest is provided.

 Also, bad hearing is an exclusion criterion but one of the participants had trouble performing the test due to hearing difficulties. Why was this patient included regardless?

 In sum, I would like to encourage Webb et al. to elaborate on and show that these issues relating to the sample had no effect and/or add these points on the risk of sampling bias and sample characteristics to their limitation section.

 Furthermore, I consider the simultaneous validation of two different forms of presentation (telephone or video conference) within one study to be unfavourable. As described in the study, the two forms of presentation place different demands on the patients and their environment. It is very patient-friendly to adapt the implementation to the preferences of the individual, but in my view, it also distorts the result. In the case of the present study, not only was the basic decision for one of the methods discussed with the patients, but also throughout the course of the test patients switched between video conferencing and telephone administration. Thereby, changing the way of administering a rather visually based test from a visual form of administration (video conferencing) to a purely auditory delivery form does not really evaluate the same test material due to differences in the required instructions and materials in my eyes. Webb et al., for example, report different instructions that the participants received during the implementation.

 In order to give a clear picture of the strengths of the remote-OCS, a randomised allocation of the patients to a fixed form of administration would benefit the results. Particularly, since the two different forms of implementation also place different demands on the participant as well as the person administering the test. Here, too, standardisation would be interesting for the sake of comparability. In this way, not only a differentiated statement could be made about the difficulties of implementing the test via telephone vs videocall, but also its strengths could be recognised and weighed against each other.

***Analyses***

 I was surprised to see the main preregistered hypothesis and analyses revolving around the intraclass correlation coefficient (ICC) as the measure of choice, given that the original OCS validation paper has already shown issues between the parallel versions. If I understand this argument correctly, this ICC problem had already been known to the authors before registering the present study’s analysis plan. Then switching their analysis approach entirely may be acceptable, if they also report the originally planned analyses in their supplementary materials at least. However, the mentioned supplementary materials folder on the OSF appears empty. Please double-check this folder.

 Further the manuscript does not specify which form of the ICC was calculated before discarding this approach. considering a lower ICC bound of .5 to be enough for validating test-retest consistency appears rather low for such an established test, as it only indicated moderate reliability. Most importantly, changing their analysis approach also invalidates the power analysis performed for an ICC analysis. Taken together, I would encourage the authors to elaborate on their rationale and provide actual results to the reader for transparency.

 One of the main motivations for the OCS was to avoid overall binary classifications as in impaired vs unimpaired. However, the new analysis that aims to convince the reader of the tele-OCS’s validity relies purely on these binary classifications as the key metric, albeit for subtests. This approach discards much information on actual test-retest scores, even if it is subtle information, which the actual raw scores could provide. Please consider reporting these scores.

 Even more concerning is the author’s reliance on binary impairment classification and norms from the in-person test due to a lack of tele-OCS data. This point is being acknowledge as a major limitation by the authors themselves. This raises the question of the validity of their analysis used for making their claims of having successfully validated the tele-OCS. Once again, it could be addressed by collecting a larger sample and adapting the analysis.

 Lastly, the authors switched in their analysis towards using several t-tests and effect sizes for their claims on (non-)significant differences. However, these results may suffer from two issues. First, multiple t-tests come with the problem of an increased false positive rate, as the authors tested data from the same participants. Please report whether these tests were corrected for multiple comparisons. Second, in the context of this investigation no difference (i.e., evidence for the null hypothesis) is the desired test outcome. T-tests cannot provide this information. Therefore, I’d suggest switching from t-tests to BayesFactors for being able to report the evidence for the null hypothesis directly.

***Figures***

 The figures are missing elaborate figure captions that explain all aspects of the figure. For example, the impaired and unimpaired labels and the fact that some bars appear to be missing for some tests, which is simply due to the value being 0. Relating to my previous point on plotting all the raw scores, here, I’d suggest plotting the continuous data as raincloud (https://doi.org/10.12688/wellcomeopenres.15191.2), violin or similar plots and avoid barplots in order to provide the reader with additional, valuable information and thereby facilitate trust in their interesting results.

**Minor Issues**

 Information on the test’s delivery as part of the procedure section could be enhanced by providing the links to the online videos hosted on Youtube ( https://www.youtube.com/watch?v=CC_QVJHqFvk) or the videos could be uploaded to the OSF repository.

Is the work clearly and accurately presented and does it cite the current literature?

Yes

If applicable, is the statistical analysis and its interpretation appropriate?

Partly

Are all the source data underlying the results available to ensure full reproducibility?

Yes

Is the study design appropriate and is the work technically sound?

Partly

Are the conclusions drawn adequately supported by the results?

No

Are sufficient details of methods and analysis provided to allow replication by others?

Yes

Reviewer Expertise:

Cognitive neuroscience and psychology, psychiatry, remote assessments

We confirm that we have read this submission and believe that we have an appropriate level of expertise to confirm that it is of an acceptable scientific standard, however we have significant reservations, as outlined above.

## References

[1] : 12-Monats-Prävalenz einer koronaren Herzkrankheit in Deutschland. *Journal of Health Monitoring* .2017; 10.17886/RKI-GBE-2017-009

[2] : Global Epidemiology of Stroke and Access to Acute Ischemic Stroke Interventions. *Neurology* .2021;97(20 Suppl 2) : 10.1212/WNL.0000000000012781 S6-S16 10.1212/WNL.0000000000012781 34785599

# Response

**Published:** 2024-03-15

**Authors:** Sam Webb

**Affiliations:** University of Oxford, UK

Title: ‘Introducing the Tele-OCS: Preliminary evidence for a remotely administered version of The Oxford Cognitive Screen’  

**Response to reviewers** We thank the reviewers and the editors for providing feedback on this manuscript draft. Please find below a detailed response to each reviewer comment.   Reviewer comments are in italics, our reply is in bullet point format in plain text, and all changes made in the manuscript text are in red.  

**Reviewer 1:** ***Webb et al. attempt to provide an important development of the OCS by attempting to validate the remote version of the Oxford Screening Tool administered via telephone or videocall. The Covid-19 pandemic has shown us that many studies and assessments can also be delivered online providing somewhat comparable results. The benefits of remote assessments and studies are undisputed. However, remote delivery comes with considerable challenges including much reduced control over the test administration and testing environment. This potential pitfall is exacerbated when delivering an assessment via telephone to potentially cognitively impaired patients. In the present manuscript, by postulating similar impairment classification of the same participants even under changed conditions, Webb et. al. test their remote OCS version and conclude it to be a valid version across telephone and videocall delivery channels. While I appreciate their open science practices, particularly their sampling and testing practices come with a number of considerable shortfalls that do not allow for drawing the all-encompassing conclusion of the remote OCS being a widely validated test after this rather small sample study. I will elaborate on and discuss these points in more detail below.*** **Major Issues** **Recruitment and sample characteristics** **1) *With their study to validate the remotely administered oxford cognitive screen (OCS), Webb et al. attempted a previously analogue assessment of cognitive performance. One crucial issue of the study lies in the recruitment of participants. The authors used the strategy of community dwelling to recruit their participants but these individuals were sourced from completed studies of the Oxford Cognitive Screening Programme or other studies which tested or trained the cognitive performance of patients after a stroke. In my opinion, this creates a recruitment bias. Due to the prior participation in other studies, whereby the selection of participants is not at random and thus the sample is not representative of the underlying population. As well, since patients were recruited as part of a 6 month follow up and also after other studies, it would be important to clarify whether these participants had already done the OCS at least once within the first months after their stroke, as I presume the potential for practice effects exists. However, this piece of information is not provided. This could be tested by comparing reliability between participants who had taken the test before starting this study and those who have not. Similarly, the existing study design results in an increased risk of selection bias, as only participants who completed the previous studies were included. If one recruits only from the group of test persons who have already completed a study, one may suspect that these are particularly motivated and performance-oriented individuals. Hence, generalisability to the general population may not be given.***  

- We thank the reviewer for their comments. With regards to your comment on potential for practice effects due to repeated exposure to the OCS, within Oxfordshire all stroke survivors would have at least attempted the OCS as part of their routine care, as this is recommended standard practice in the UK (Royal College of Physicians, 2016). Because of this, it would be difficult to recruit stroke survivors within Oxfordshire who had not completed the OCS beforehand and therefore comparing reliability between participants who had completed the OCS prior to the current study would not be meaningful.  We have made this point more clearly as below

--- We used a prospective cross-sectional within-subjects design. Due to the standard practice of in stroke care within Oxfordshire, most stroke survivors admitted to a hospital for stroke will complete, or partially complete, the OCS. We recruited chronic stroke survivors in the UK between November 2021 to August 2022. All participants completed the OCS when originally admitted to hospital, separate to this study.   ---  

- We also note this as a limitation in our discussion.

  --- Finally, all participants were previously exposed to the OCS, whether as part of our research, or as part of their admission to hospital where OCS is given as standard where it can be. This means, there may be a chance for practice effects. We note, we did not observe many in the current study, so it is reasonable to assume less exist between first exposure and the current study. Alternatively, due to previous exposure, participants may be better at the OCS in general, which would explain our low rates of impairment. As other studies using the OCS have found impairment rates, even for those previously exposed, and at different time points post-stroke (Kusec *et al*., 2023; Milosevich *et al*., 2023), we do not feel this is a major issue in the current study.   ---  

- With regards to your comment on potential risk of recruitment and selection bias, both OCS-Recovery or OX-CHRONIC studies had broad inclusion criteria (i.e., diagnosis of a stroke, age >18, ability to provide informed consent, ability to concentrate for at least 20 minutes) to maximise those who are recruited. Due to this, there are few barriers to participation and enhanced our sample representativeness. As reflected in our demographics, we have recruited a representative pool of participants from the Oxfordshire area, reflecting the typical age, education, and ethnicity of stroke survivors, as well as stroke hemisphere, and stroke severity. We note we also well reflected the ethnicity and age statistics of older stroke survivors, whereby most (>95%) of stroke survivors are white and white-British in the UK if they are over 65 (Census data 2021, older persons report, see https://www.ons.gov.uk/peoplepopulationandcommunity/birthsdeathsandmarriages/ageing/articles/profileoftheolderpopulationlivinginenglandandwalesin2021andchangessince2011/2023-04-03 ),

 

- We acknowledge that, as in any research study, potential for selection bias exists. We have expanded on this in the discussion as below:

---

- We used a prospective cross-sectional within-subjects design. Due to the standard practice of inpatient stroke care within Oxfordshire, most stroke survivors admitted to a hospital for stroke will complete, or partially complete, the OCS, prior to commencing any research study. We recruited chronic stroke survivors in the UK between November 2021 to August 2022. All participants completed the OCS when originally admitted to hospital, separate to this study. We contacted long-term stroke survivors from the OX-CHRONIC (Kusec et al., 2023) study who had recently (within 30 days at time of contact) completed the Tele-OCS as part of the OX-CHRONIC protocol. Additionally, we asked all participants from the Oxford Cognitive Screening programme, who had just (at time of being contacted) completed an in person 6-month follow up OCS (Demeyere *et al.*, 2015; Milosevich, Moore, Pendlebury, & Demeyere, 2023), to take part. We aimed to get both the OCS and Tele-OCS completed within 30 days of each other, but this was not always possible. Inclusion criteria for both the Oxford Cognitive Screening programme and OX-CHRONIC are very inclusive and aligned with the current inclusion criteria. This means the sampling approaches originally used have minimal bias (except bias of those who are willing to participate), and the underlying samples are representative of the UK stroke population. In the current study we attempted to reflect the UK stroke population in key demographics of age, education, sex, stroke type, stroke side, and stroke severity.

--- from discussion Moreover, it is necessary to consider that those who consent to take part in research may be different from those who do not, and as such more inclusive study protocols using those with in-capacity to consent to research may help contribute to more reflective demographics. ---  

***2)         As previously mentioned, the Information regarding the participants is rather limited. Webb* *et al. reported most participants being white males as a weakness of the study. They did also not address the presence of ischemic strokes either, which account for 70% of their sample. This is much lower than the prevalence reported in previous studies of around 87% (Busch & Kuhnert, 2017; Saini et al., 2021), which may affect generalisability.*** ***Following on from this point, generalisability to the entire British population or beyond and to various remote delivery channels is also questionable, given the small sample (N=40), the heavy reliance on white British males and most of these participants taking the test on the telephone. To warrant the authors claims of presenting a successfully validated test, the sample size would need to be increased and sample’s characteristics more balanced. In sum, I would like to encourage Webb et al. to elaborate on and show that these issues relating to the sample had no effect and/or add these points on the risk of sampling bias and sample characteristics to their limitation section.***  

- We thank the reviewer for their comments. We have now restructured our limitations section to elaborate on the homogeneity of our sample. We note that we have already called for larger studies, but have specifically edited this now (see next with changes in red:

--- Although our study sample was statistically powered, the sample size was small and relatively homogeneous. For example, 97.5% of the sample was White-British, 70% were male, and around 70% were ischaemic stroke. Ideally, there would be around 82% White-British participants to reflect UK government 2021 census data for ethnicity (ONS, 2022), or >93% for older adults specifically, or 87% for general UK stroke survivors (Douiri et al., 2021), approximate equality of males and female and around 87-90% ischemic stroke  (Douiri et al., 2021; King *et al.*, 2020). Strengths of our sample, however, include age and stroke severity, which represent the general UK stroke population (Douiri et al., 2021). Furthermore, given that many participants were in the chronic stage of stroke, they were often classified as unimpaired across subtasks in both modalities, limiting our interpretation for stroke survivors with more severe impairments. This may impact the generalisability of the present results to the wider stroke population. Although the majority of stroke survivors tend to be males, our weighting of males to females was biased as we had far more males. This may impact the generalisability of our results to females. We do not feel that the gender balance in our data impacts the results drastically, however, we acknowledge this as a limitation. We did not choose to purposively sample for sex specifically as it is not thought to alter the results of global cognitive scoring after a stroke. Future validation studies of the Tele-OCS may benefit from using larger, more heterogeneous samples, including those who have sustained more severe strokes, and more representative demographics.   Finally, all participants were previously exposed to the OCS, whether as part of our research, or as part of their admission to hospital where OCS is given as standard where it can be. This means, there may be a chance for practice effects. We note, we did not observe many in the current study, so it is reasonable to assume less exist between first exposure and the current study. Alternatively, due to previous exposure, participants may be better at the OCS in general, which would explain our low rates of impairment. As other studies using the OCS have found impairment rates, even for those previously exposed, and at different time points post-stroke (Kusec *et al*., 2023; Milosevich *et al*., 2023), we do not feel this is a major issue in the current study. ---

***3)         It may also come with different symptoms compared to the presence of subarachnoid and intracerebral haemorrhages. A more precise, detailed presentation of the included participants would be desirable for the reader.***  

- Due to (sub) sample size, evaluating performance differences by stroke subtype or other demographics was not considered meaningful. We have noted this potential sample bias in the limitations section. We regret to not have further details available for the sample. We have added acute OCS data which was collected prior to the study from participant medical files. We have now included performance on the OCS subtasks in the form of mean, SD, and range to show the range of performance, to reveal potential biases. See supplemental table S1 and extract from the data analysis section below:

--- Data analysis Prior to formal analyses we examined sample characteristics, including demographics and prior OCS performance taken within 20 days of stroke. Acute OCS performance is presented in Table S2 in the supplementary materials. ---    

***4)         Further, the authors do not address the interval between the stroke and the time of the assessment. Here, too, a more detailed description and analysis would be desirable due to the following reasons. The time post-stroke in which participants took part varied largely. The IQR suggests that some had a twice as long (or an even longer) recovery period before they participated in the study. Some participants were included after more than a 6.5 times longer recovery time (1500/228 days) compared to the median. Since the study design was within-subjects this aspect may not matter too much by itself but contributes to much heterogeneity in recovery possibilities within an already small sample.***  

- Given the Tele-OCS is intended to be used in community-based samples, we did not aim to recruit participants from a specific time post-stroke, and instead capitalised on existing data (to reduce participant burden) from projects comprising stroke survivors at both 6-months to > 2 years post-stroke. To clarify this, we now note in the participant recruitment section that we recruited chronic stroke survivors, see below from participants section

--- We used a prospective cross-sectional within-subjects design. Due to the standard practice of in stroke care within Oxfordshire, most stroke survivors admitted to a hospital for stroke will complete, or partially complete, the OCS. We recruited chronic stroke survivors in the UK between November 2021 to August 2022. All participants completed the OCS when originally admitted to hospital, separate to this study. We contacted long-term stroke survivors from the OXCHRONIC (Kusec et al., 2023) study who had recently (within 30 days at time of contact) completed the tele-OCS as part of the OXCHRONIC protocol. The OXCHRONIC population were at minimum 2 years post-stroke with an average of 4.5 years. Additionally, we asked all participants from the Oxford Cognitive Screening programme, who had just (at time of being contacted) completed an in person 6-month follow up OCS (Demeyere *et al.*, 2015; Milosevich, Moore, Pendlebury, & Demeyere, 2023), to take part. We aimed to get both the OCS and Tele-OCS completed within 30 days of each other, but this was not always possible. ---  

- To investigate whether time post-stroke had any detectable effect on performance, we conducted a correlation analysis between time post-stroke and proportion of OCS subtasks impaired, in which no significant relationship was found (copied below):

--- The time in days between in-person and Tele-OCS administration ranged from 6 to 72, with an average of *M*=27.70 days ( *SD*=12.29). 23 participants were administered the Tele-OCS first (57.5%). Time interval was not correlated with any differences in subtasks impaired on either modality ( *r*(38)= -0.03, *p*=.85). Administration of the Tele-OCS took an average time of 17.17 minutes ( *SD*=2.73, range = 14 - 22). The Tele-OCS was administered using phone to the majority of participants ( *n*=36, 90%). ---

- As presented in our results, there were limited cases of trends in improvement in across modality, meaning it is unlikely that there would have been recovery in between assessment time points.  

   
***5)         Following on from this, the within-subject test-retest interval between the in-person and remote tests shows some considerable range as well. Here, I’m doubtful that a gap of 6 days is comparable with one of 72 days. Hence, I’d suggest to the authors to ensure that participants with a shorter interval did not show more consistency in their results compared to those with an interval longer than the mean. This could also be tested using a correlative approach correlating test-retest interval and the difference in % of the proportion of subtasks impaired.***    

-     see figure 1 linked here: https://healthopenresearch.s3.eu-west-1.amazonaws.com/linked/182865.13291-_comments_image.pdf
- We thank the reviewer for this comment highlighting that there may be differences in performance for those with shorter vs longer assessment intervals. In investigating this relationship, we found no correlation between the difference in proportion of tasks impaired and the time between assessments (r(38)= -0.03, *p*=.85). We have additionally plotted this correlation, with proportion of tasks impaired on the y-axis, and time between sessions on the x axis, indicating no clear pattern of performance across time intervals.

     
***6)         The inclusion/exclusion criteria of the study were described by Webb et al. but appear to miss some important information. On the one hand, participants have a history of a confirmed stroke, but the severity or location of the stroke is not described. May this have an effect?***   Stroke location (hemisphere) and stroke severity are detailed in Table 1. We do not have immediate access to scan data as this was not part of our protocol, so we cannot report on exact location of stroke.  We separately examined the effect of sex, stroke type, and stroke hemisphere on subtasks impaired on the OCS across modality and found no significant main effect or interaction of demographics on performance. Results are detailed in the supplementary and references now in text.  

***7)         I would also like to see the authors provide a more detailed description of the included participants and how their characteristics relate to the results, as no information on relevant symptoms such as neglect or hemiparesis among the participants other than may be indirectly obtained from the broken hearts subtest is provided.***  

- We unfortunately did not collect information for characteristics such as hemiparesis. Neglect among stroke survivors in the Oxfordshire area is tested with the Broken hearts task, and can thus be used to determine the rate of impairment in our sample. However, we feel our sample is too small for further subgroup analyses of different sub-categories of participants.
- We have now better described the sample and provided prior OCS performance scores for reference. It can be seen via the range of scores that impairments in scores were seen throughout the OCS subtasks.

 
***8)         Also, bad hearing is an exclusion criterion but one of the participants had trouble performing the test due to hearing difficulties. Why was this patient included regardless?***

- This exclusion criteria refer to hearing difficulties that could not be reasonably adjusted for. Any hearing difficulties that became evident whilst testing were overcome by repeating a single instruction twice. To clarify this point, we have stated in the manuscript:

--- Participant identification Eligibility criteria were: 1) history of confirmed stroke; 2) 18 years of age or older at time of stroke; 3) capacity to consent to research; 4) able to remain alert for at least 20 minutes; and 5) spoke and understood sufficient English. Participants were excluded if they had hearing, language, or visual impairments (not including visual neglect) that would not allow for remote or in-person assessment outside of reasonable adjustments. Any hearing difficulties that became evident whilst testing were overcome by repeating a single instruction twice. ---      

***9)         Furthermore, I consider the simultaneous validation of two different forms of presentation (telephone or video conference) within one study to be unfavourable. As described in the study, the two forms of presentation place different demands on the patients and their environment. It is very patient-friendly to adapt the implementation to the preferences of the individual, but in my view, it also distorts the result.***

- We agree with the reviewer that including different remote modalities (i.e., telephone vs in person) may be a weakness of the paper because we do not directly test their equivalence (nor did we aim to) due to low numbers in any comparison. However, the administration protocol and materials are identical for each remote modality, as detailed in the Tele-OCS description within the manuscript. When the Tele-OCS is conducted via videoconferencing, there are no additional demonstrations or instructions, therefore any potential differences are not currently testable. Participant preference of remote modality in our study is reflective of using the Tele-OCS in clinical settings where choice of modality would be offered.
- We acknowledge that differences in remote modality should be investigated in future studies within our limitations, as below ):

--- There is currently no normative data for performance on the Tele-OCS for comparison of scores, as our results rely on the in-person normative data to generate impairment classifications we cannot know if these impairment classifications are accurate. Further research will need to determine this. Furthermore, a small part of our study concerned different modalities of assessment (telephone vs videocall). We did not aim to assess whether the modality of remote assessment affected performance, as we relied on participant preference above all else, had too small a sample to do so, and we feel that as the instructions and materials are identical, there should be limited differences. ---

***10)       In the case of the present study, not only was the basic decision for one of the methods discussed with the patients, but also throughout the course of the test patients switched between video conferencing and telephone administration. Thereby, changing the way of administering a rather visually based test from a visual form of administration (video conferencing) to a purely auditory delivery form does not really evaluate the same test material due to differences in the required instructions and materials in my eyes. Webb et al., for example, report different instructions that the participants received during the implementation.***   Apologies this was not transparent, to be clear:

- There is no case reported of any participants switching modalities within a session, all were consistent.
- As noted in our response above, administration of the Tele-OCS both via telephone and videoconferencing have identical instructions and testing materials.
- We have now added some clarification to the manuscript, see below:

--- Procedure Once participants from the Oxford Cognitive Screening programme were identified as having just (within 30 days) completed an in-person OCS in their homes, we asked if they would complete the Tele-OCS. For those who agreed, we posted the Tele-OCS pack at least 1 week before the appointment and reminded them not to open the pack until instructed. In the session, informed consent was taken and the Tele-OCS was administered either by phone or videoconferencing depending on the preference of the participant. Shifting modality during a session was not permitted, nor did this come up during data collection. We provided a pre-paid envelope for participants to post their packs back. If participants were recruited from the OXCHRONIC study, we contacted them within 30 days of having completed the Tele-OCS for the OXCHRONIC protocol, and administered the OCS in person at their homes. ---      

***11)       In order to give a clear picture of the strengths of the remote-OCS, a randomised allocation of the patients to a fixed form of administration would benefit the results. Particularly, since the two different forms of implementation also place different demands on the participant as well as the person administering the test. Here, too, standardisation would be interesting for the sake of comparability. In this way, not only a differentiated statement could be made about the difficulties of implementing the test via telephone vs videocall, but also its strengths could be recognised and weighed against each other.***  

- We thank the reviewers for this insight and agree that it may be worthwhile to randomise the allocation of participants to telephone vs videocall modalities in future studies. This could elucidate potential differences between these types of assessment method. This has now been added to the limitations section of the discussion. However, we want to re-iterate that it was not possible to randomly allocate participants to modalities in the current study for a number of reasons (which are noted in the limitations section).
- Moreover, and more generally from the current study, randomisation may not be feasible/possible with stroke participants as some individuals do not have any familiarity with videoconferencing/do not have access to internet/restricted internet in their geographical area. Additionally participants may have smart phones but may not a tablet or computer to complete the assessment in the same way - unless you recruited people who can do both at the outset (but this would likely result in greater bias in the sample).
- Changes to the manuscript discussion are in red:

--- There is currently no normative data for performance on the Tele-OCS for comparison of scores, as our results rely on the in-person normative data to generate impairment classifications we cannot know if these impairment classifications are accurate. Further research will need to determine this. Furthermore, a small part of our study concerned different modalities of remote assessment (phone vs videocall). We did not aim to assess whether the modality of remote assessment affected performance, as we relied on participant preference above all else, had too small a sample to do so, given identical instructions and materials, there should be limited differences. Furthermore, it should be noted that stroke survivors do not always have the correct equipment to carry out assessment via videoconferencing. However, it may be worthwhile for future studies with larger sample sizes to randomly allocate participants to either telephone or videoconferencing to investigate whether there are any differences between these types of remote assessment methods. ***---***

***Analyses*** ***12)       I was surprised to see the main preregistered hypothesis and analyses revolving around the intraclass correlation coefficient (ICC) as the measure of choice, given that the original OCS validation paper has already shown issues between the parallel versions. If I understand this argument correctly, this ICC problem had already been known to the authors before registering the present study’s analysis plan. ***

- Our original ICC analysis were pre-registered as they were an appropriate test to use for continuous subtask scores in theory, however, the small ranges of variation observed within our sample precluded the use of ICCs in a sensible manner. Therefore, it was the high concordance between time points in our observed data, rather than the OCS subtests itself, that meant we had to switch analysis plan. This has now been clarified in the paper as below:

--- This project was originally pre-registered at the start of data collection (3 participants had taken part). The preregistered analysis plan was inspired by ICCs between OCS parallel versions A and B in the original OCS normative article (Demeyere *et al*., 2015), though once data was collected, it became clear that this was not the best approach for the current data that was observed. The small variation of scores observed in the subtasks meant the raw continuous scores ICCs between in-person and remote subtest comparisons were not appropriate. We instead opted to shift towards test of difference between modalities, to retain the consistent statistical power. The sample size calculations remained the same as we were similarly powered for equivalent comparisons via difference based *t*-tests. ---  

***13)       Then switching their analysis approach entirely may be acceptable, if they also report the originally planned analyses in their supplementary materials at least. However, the mentioned supplementary materials folder on the OSF appears empty. Please double-check this folder.***

- Our apologies for the oversight in not uploading the original planned analyses within our supplementary materials. Supplementary materials are now uploaded to OSF, which include the results of the ICCs mentioned in the main manuscript (and the underlying code to reproduce them).

 

***14)       Further the manuscript does not specify which form of the ICC was calculated before discarding this approach.***

- We thank the reviewer for pointing out this oversight which has now been corrected both in the manuscript and in supplemental materials.

--- Sample size was pre-determined in our pre-registration at a minimum of 30 participants to detect a two-way random effects single unit agreement based intraclass correlation coefficient (ICC) ---  

***15)       Considering a lower ICC bound of .5 to be enough for validating test-retest consistency appears rather low for such an established test, as it only indicated moderate reliability. Most importantly, changing their analysis approach also invalidates the power analysis performed for an ICC analysis. Taken together, I would encourage the authors to elaborate on their rationale and provide actual results to the reader for transparency.***  

- We agree that the manuscript would benefit from providing greater information power for the adjusted analyses and on our decisions for a sufficient ICC for the Tele-OCS. We firstly note that there was little difference in the power analysis for sample size for a t-test or ICC for this study, but this has now been clarified in our manuscript:

--- Changes from pre-registration This project was originally pre-registered at the start of data collection (3 participants had taken part). The preregistered analysis plan was inspired by ICCs between OCS parallel versions A and B in the original OCS normative article (Demeyere *et al*., 2015), though once data was collected, it became clear that this was not the best approach for the current data that was observed. The small variation of scores observed in the subtasks meant the raw continuous scores ICCs between in-person and remote subtest comparisons were not appropriate. We instead opted to shift towards test of difference between modalities, to retain consistent statistical power. The sample size calculations remained the same as we were similarly powered for equivalent comparisons via difference based *t*-tests. We present ICC analyses in extended data. ---  

- Regarding our decision to have a lower bound of 0.50 ICC, this was considered to be the minimum acceptable ICC for the Tele-OCS and not the expected ICC estimate. As you have noted, this lower bound would reflect moderate reliability and our study was powered to detect this.

 
***16)       One of the main motivations for the OCS was to avoid overall binary classifications as in impaired vs unimpaired. However, the new analysis that aims to convince the reader of the tele-OCS’s validity relies purely on these binary classifications as the key metric, albeit for subtests. This approach discards much information on actual test-retest scores, even if it is subtle information, which the actual raw scores could provide. Please consider reporting these scores.***

- We agree that a strength of the OCS is that it provides insight into domain-specific  strengths and weaknesses of the stroke survivors being assessed. The OCS was developed to have impairment classifications per subtask, as this is how it would be used in clinical settings to determine domain-specific impairments. The original OCS aims to classify impairment based on specific cognitive domains, rather than providing a single domain-general impairment classification that provides limited information. In this sense, the OCS and the Tele-OCS provide more granular information about cognition that is used pragmatically in clinical settings. In Table 2,  we have also presented raw data on continuous scores in the manuscript. As stated in the paper, the use of impairment classifications is more in line with both the aims of the OCS, and with how the OCS is used in practice.

 
***17)       Even more concerning is the author’s reliance on binary impairment classification and norms from the in-person test due to a lack of tele-OCS data. This point is being acknowledge as a major limitation by the authors themselves. This raises the question of the validity of their analysis used for making their claims of having successfully validated the tele-OCS. Once again, it could be addressed by collecting a larger sample and adapting the analysis.***   We agree with these comments and await further research about the generation of  new normative scores for the Tele-OCS. We have updated the text throughout as well as the title of the manuscript to refer to “Preliminary evidence” and also discuss this explicitly in the limitations.    

***18)       Lastly, the authors switched in their analysis towards using several t-tests and effect sizes for their claims on (non-)significant differences. However, these results may suffer from two issues. First, multiple t-tests come with the problem of an increased false positive rate, as the authors tested data from the same participants. Please report whether these tests were corrected for multiple comparisons. ***

- We thank the reviewer for identifying this oversight. In Table 2 where we report Cohen’s d between OCS and Tele-OCS subtasks, we have now added a note to our table legend indicating the corrected alpha, as below:  

[i] Note. ‘*’ signifies p-value <.05. Multiple comparison alpha corrected level is .005 (.05/11 = .005). ‘**’ signifies p-values <.005).  

***19)       Second, in the context of this investigation no difference (i.e., evidence for the null hypothesis) is the desired test outcome. T-tests cannot provide this information. Therefore, I’d suggest switching from t-tests to BayesFactors for being able to report the evidence for the null hypothesis directly.***

- Thank you for your comments, we have now included equivalence tests in addition to t-tests to account for different interpretations. Initially we wished to evaluate any differences in performance, hence our decision to use t-tests. We have now included whether the differences are smaller than the smallest effect size we can detect.  The results of our equivalence tests (presented in the *Supplementary Materials) *are in line with the t-test results. For consistency with the remainder of the manuscript and literature on the OCS, we have opted for frequentist statistics which are more understandable by the general scientific community, and equivalence tests here (and t-test with our original aim) are appropriate to answer our questions.

  In Table 2, we present descriptive statistics for raw score performance on each shared OCS subtask as well as repeated-measures Cohen’s *d* and significance of difference on *t*-tests between each subtask. Equivalence tests were additionally conducted, paralleling the t-test results presented here (see Supplementary Materials).  

***Figures*** ***20)       The figures are missing elaborate figure captions that explain all aspects of the figure. For example, the impaired and unimpaired labels and the fact that some bars appear to be missing for some tests, which is simply due to the value being 0. Relating to my previous point on plotting all the raw scores, here, I’d suggest plotting the continuous data as raincloud (https://doi.org/10.12688/wellcomeopenres.15191.2), violin or similar plots and avoid barplots in order to provide the reader with additional, valuable information and thereby facilitate trust in their interesting results.***

- We agree that more detailed captions were needed and have now elaborated on them to improve ease of understanding.
- Prior to initial submission, we attempted raincloud plots but these became difficult to read when clustered together as a single figure, and to visually compare performance of the OCS to the Tele-OCS. We therefore chose to use density distributions to appropriately characterise distributions of data and overlap between in-person and remote administrations of the OCS. We hope this is acceptable. See plot: see figure 2 linked here: https://healthopenresearch.s3.eu-west-1.amazonaws.com/linked/182866.13291-_comments_image_2.pdf

                     
***Minor Issues*** ***21)       Information on the test’s delivery as part of the procedure section could be enhanced by providing the links to the online videos hosted on Youtube (https://www.youtube.com/watch?v=CC_QVJHqFvk) or the videos could be uploaded to the OSF repository.***

- There are currently no videos which reflect the administration of the Tele-OCS, videos that do exist were created in 2020 as part of our interim remote OCS advice. These are being developed.
